# Coding Properties of Mouse Retinal Ganglion Cells with Dual-Peak Patterns with Respect to Stimulus Intervals

**DOI:** 10.3389/fncom.2016.00075

**Published:** 2016-07-19

**Authors:** Ru-Jia Yan, Hai-Qing Gong, Pu-Ming Zhang, Pei-Ji Liang

**Affiliations:** School of Biomedical Engineering, Shanghai Jiao Tong UniversityShanghai, China

**Keywords:** retinal ganglion cell, dual-peak response, response latency, spike count, linear discriminant analysis, information coding, stimulus interval

## Abstract

How visual information is encoded in spikes of retinal ganglion cells (RGCs) is essential in visual neuroscience. In the present study, we investigated the coding properties of mouse RGCs with dual-peak patterns with respect to visual stimulus intervals. We first analyzed the response properties, and observed that the latencies and spike counts of the two response peaks in the dual-peak pattern exhibited systematic changes with the preceding light-OFF interval. We then applied linear discriminant analysis (LDA) to assess the relative contributions of response characteristics of both peaks in information coding regarding the preceding stimulus interval. It was found that for each peak, the discrimination results were far better than chance level based on either latency or spike count, and were further improved by using the combination of the two parameters. Furthermore, the best discrimination results were obtained when latencies and spike counts of both peaks were considered in combination. In addition, the correct rate for stimulation discrimination was higher when RGC population activity was considered as compare to single neuron's activity, and the correct rate was increased with the group size. These results suggest that rate coding, temporal coding, and population coding are all involved in encoding the different stimulus-interval patterns, and the two response peaks in the dual-peak pattern carry complementary information about stimulus interval.

## Introduction

Visual information is transmitted to the brain by spike trains of retinal ganglion cells (RGCs) (Masland, 2001). How spike trains from RGCs represent the visual world is one of the central issues in the field of visual neuroscience. It has long been assumed that information about visual stimuli is carried by the time-varying firing rates of RGCs according to the work of Lord Adrian (Adrian and Zotterman, 1926). However, in recent years, the importance of spike patterns for neural coding has been receiving increasing attention (Berry et al., 1997; Lesica and Stanley, 2004; Greschner et al., 2006; Gong et al., 2010). Several previous studies showed that different RGCs exhibited different spike patterns in response to the same stimulation, and may employ different coding strategies and play different roles in information transmission (Xu et al., 2005; Gollisch and Meister, 2008). Among the RGCs, some respond rapidly at stimulus onset with relatively high firing rate (“brisk”), while others respond with relatively longer latency and lower rate (“sluggish”) (Cleland and Levick, 1974). Brisk cells transmit information at higher rates but with similar efficiency as compared to sluggish cells (Koch et al., 2004, 2006).

In response to spatially homogeneous light flashes, a particular pattern termed “dual-peak” response has been observed. The dual-peak response consists of two response components: a transient component occurring within a short interval (50–100 ms) relative to the stimulus onset, and another following component occurring tens to hundreds of milliseconds after the initial one, thus resulting in two peaks in the peri-stimulus time histogram (PSTH) (Soucy et al., 1998; Segev et al., 2006; Thiel et al., 2006; Zhou et al., 2007; Yan et al., 2016), possible generating mechanism for this particular pattern was also suggested (Yan et al., 2016). However the coding properties of dual-peak pattern, particularly the contribution of the second peak to stimulus information coding, still remained largely unknown.

Stimulus identification is an important function of the nervous system, and at the meantime, stimulus discrimination performance also provides an approach for analyzing neuronal coding properties (Kenyon et al., 2004; Pillow et al., 2005; Schwartz et al., 2012). The relative importances of various response parameters to the neural coding can be assessed by comparing their contributions in stimulus discrimination (Fernandez et al., 2000; Greschner et al., 2006). Response latency and firing rate are basic characteristics of RGC response, and are suggested to efficiently transmit information about stimulus features, such as stimulus wavelength, luminance, contrast, motion speed, and direction, etc. (Fernandez et al., 2000; Greschner et al., 2006; Thiel et al., 2007; Risner et al., 2010). Meanwhile, RGC population activity patterns can also vary according to stimulation properties including spatial patterns, luminance, motion direction, etc., and are also suggested to carry visual information (Ackert et al., 2006; Jing et al., 2010; Xiao et al., 2013).

Stimulus duration or interval is an important feature of visual stimulation. In the present study, dual-peak responses were observed from RGCs' ON responses. Since, the properties of ON responses were modulated by preceding light-OFF intervals (Xiao et al., 2014b), we focused on the stimulus-interval-dependent ON-response changes and information coding in RGCs with dual-peak patterns, using full-field flashes with different light-OFF intervals. The response latencies and firing rates of the two components in the dual-peak pattern were measured respectively, and they all exhibited systematic changes with the preceding light-OFF interval. Light-OFF interval was then identified based on different RGC response characteristics extracted from both single cells and RGC groups, using linear discriminant analysis (LDA). It was found that stimulus-interval patterns were better discriminated when the characteristics of both response peaks were considered for single cell and across the population. In addition, RGC groups performed better in visual discrimination than single cells, and the correct rate was positively correlated with group size. These results added to previous findings that both peaks in dual-peak pattern were involved in light intensity coding (Thiel et al., 2006), and suggest that they might carry stimulation information complementary to each other.

## Materials and methods

### Retina preparation and electrophysiological recording

Experiments were performed on isolated retinas of adult C57BL/6 mice (2–3 months). Mice were dark-adapted for 30 min prior to the experiment, and sacrificed under dim red light by cervical dislocation. The retina was isolated in oxygenated (95% O_2_ and 5% CO_2_) Ringer's solution containing (in mM): 124.0 NaCl, 2.5 KCl, 1.3 NaH_2_PO_4_, 2.0 CaCl_2_, 2.0 MgCl_2_, 22.0 glucose, and 26.0 NaHCO_3_.A small piece of retina (about 3 × 3 mm^2^) was cut and attached to a nitrocellulose filter (0.22 μm pore size, White GSWP, Millipore Corporation, Bedford, MA, USA), with photoreceptor side contacting the filter paper. The mounted retina was then placed on a piece of multi-electrode array (MEA, Multi Channel Systems MCS GmbH, Reutlingen, Germany) with the ganglion cell layer contacting the electrodes, and was continuously perfused with oxygenated Ringer's solution at 34–37°C.

The activities of neurons were recorded by the MEA which was connected to a recording system (MEA-System, Multi Channel System MCS GmbH). The MEA consisted of 60 electrodes (10 μm in diameter) arranged in an 8 × 8 matrix (leaving the four corners void). The horizontal and vertical tip-to-tip distances between adjacent electrodes were 100 μm. The raw electrode data were amplified through a 60-channel amplifier (single-ended, amplification 1200 ×, amplifier input impedance > 10^10^ Ω, output impedance 330 Ω). Signals from the selected channels were sampled at a rate of 20 kHz (MC_Rack, Multi Channel System MCS GmbH) and stored in a computer. Timing signals of visual stimuli were also recorded and stored in the computer.

Spikes from individual neurons were sorted based on principal component analysis (PCA) (Zhang et al., 2004), as well as the spike-sorting unit in the commercial software OfflineSorter (Plexon Inc., Dallas, Texas, USA). In order to get accurate data for spike train analysis, only single-neuron events clarified by both spike-sorting methods mentioned above were used for further analyses (Li et al., 2012).

All described procedures were reviewed and approved by Institutional Animal Care and Use Committee at Shanghai Jiao Tong University.

### Stimulation protocols

Light stimulus was generated from a computer monitor (Vision Master Pro 450, Iiyama, Japan) and was focused to an area of 0.9 × 0.9 mm^2^ when projected onto the retina via a lens system. Before stimulation protocols were applied, full-field sustained dim white light (0.19 cd/m^2^) was given for 30 s to adjust RGCs' sensitivities to similar levels (Liu et al., 2007).

The stimulation protocol contained repetitive full-field 1-s light-ON (0.38 cd/m^2^) stimuli separated by different light-OFF intervals (0.0 cd/m^2^). Totally 50 trials were displayed, with each trial containing three full-field 1-s light-ON stimuli led by randomized light-OFF intervals of 1, 5, and 9 s (1-s OFF/1-s ON, 5-s OFF/1-s ON, 9-s OFF/1-s ON). The arrangement of different light-OFF intervals was randomized in each trial to minimize the effect of adaptation.

### Identification criterion of dual-peak response

**Figure 1:**
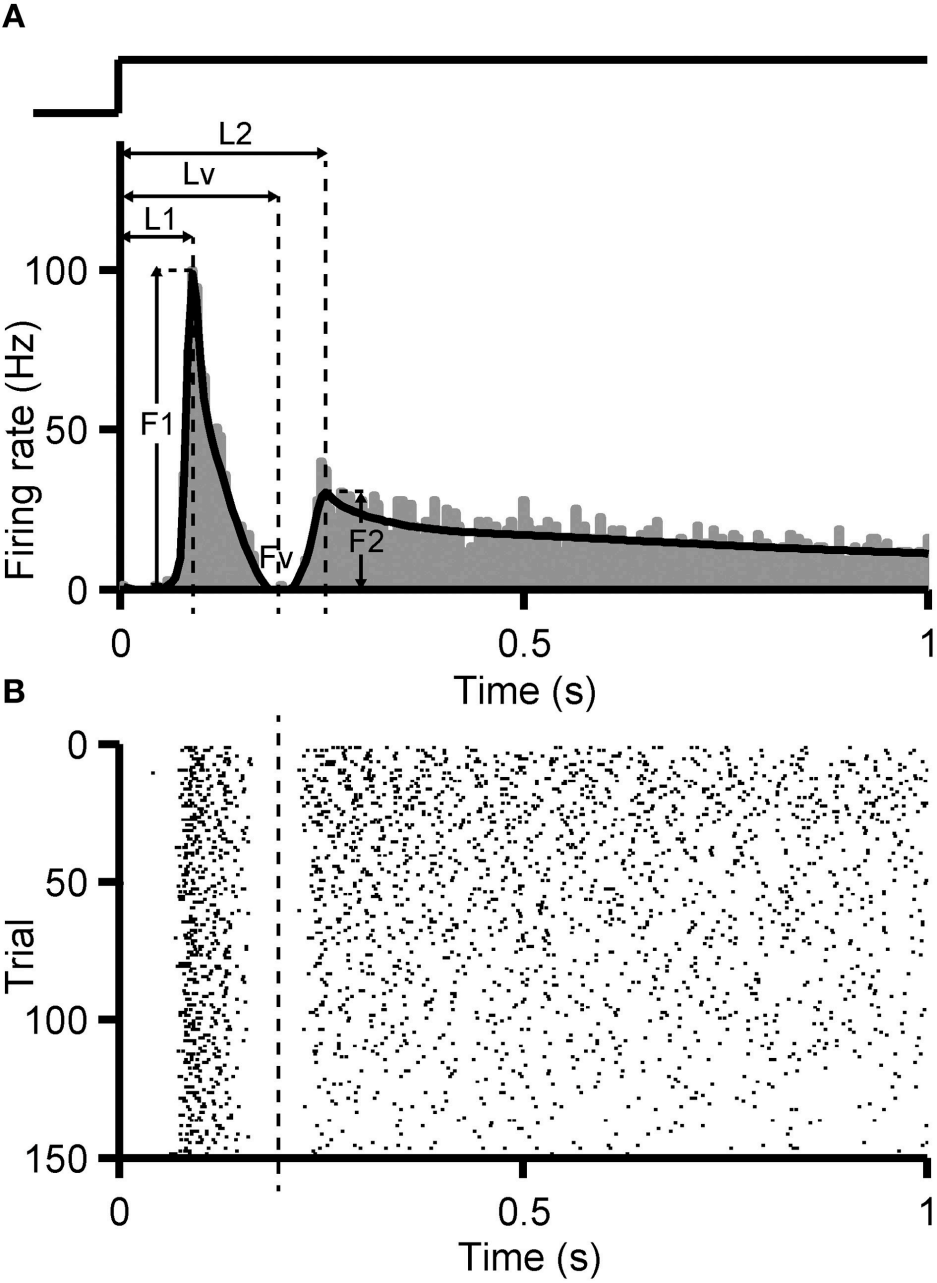
**Identification criterion of dual-peak response**. PSTH (**A**, bin size = 5 ms) and raster plot **(B)** of an example RGC with dual-peak pattern are shown. Black fitting curve superimposed on the PSTH was generated by the BARS method. F1, F2 are the peak firing rates of the first and the second peak, respectively, and Fv is the firing rate at the trough. L1, L2, Lv indicate the time gap between the stimulus onset and F1, F2, Fv, respectively. The trace above the PSTH illustrates the time course of the light stimulation.

Identification criterion of dual-peak response was set following the method introduced by Zhou et al. (2007). First, the PSTH (bin = 5 ms) of RGC's response to full-field flashes was calculated and smoothed by a non-parametric regression method Bayesian Adaptive Regression Splines (BARS) (Figure 1A, black curve) (Dimatteo et al., 2001; Kass et al., 2003). In the PSTH, if the firing rate of the first peak (F1) descends quickly and reaches a minimal level (Fv, Fv <20% F1), and a second peak rises again with peak level higher than the valley value (F2 >150% Fv, or F2 > 5 Hz when Fv = 0), a dual-peak response can be identified.

For an identified dual-peak response, the time when the trough (Lv) appeared in the PSTH was regarded as the boundary of the two response components. Every spike in each trial was assigned to a certain response peak, according to such identified boundary (Figure 1B). To investigate the stimulus-interval-dependent response changes of dual-peak RGCs, the PSTH was calculated separately for 1, 5, and 9-s/1-s (OFF/ON) stimulus patterns based on corresponding responses, and the boundary for each stimulus pattern was then identified separately.

### Linear discriminant analysis

Linear discriminant analysis (LDA) was applied to assess the ability of discriminating stimulus patterns based on different response features (Fernandez et al., 2000; Greschner et al., 2006). The basic principle of LDA method is to determine a set of linear equations performing a projection of features that minimize the within-class variance and maximize the between-class variance, and thus separate two or more classes (Jain et al., 2000; Cunningham and Yu, 2014).

Suppose there are *C* classes in a data set *X*_*ji*_ (*i* = *1, …, M*_*j*_; *j* = *1, …, C*). *X*_*ji*_ consists of features which can be used to identify the *C* classes. In the LDA calculation, each feature vector is first normalized to the range of [0, 1] using unity-based normalization before analysis (for each feature, the maximum value among all classes is set to 1, and the minimum value is set to 0) (Fard and Sadeghzadeh, 2016). Then the optimal projection direction is determined by calculating the eigenvectors of *E* = *S*${}_{w}^{-1}$*S*_*b*_, where *S*_*w*_, *S*_*b*_ are within-class scatter matrix and between-class scatter matrix, respectively (Dudoit et al., 2002; Kumar and Ravikanth, 2009):

(1) $$\begin{array}{r} S_{\text{w}}=\overset{C}{\underset{j\;=\;1}{\sum }}\overset{M_{j}}{\underset{i\;=\;1}{\sum }}\left(x_{ji}-\mu_{j}\right){\left(x_{ji}-\mu_{j}\right)}^{T},\mu_{j}=\frac{1}{M_{j}}\overset{M_{j}}{\underset{i\;=\;1}{\sum }}x_{ji} \end{array}$$

(2) $$\begin{array}{r} S_{b}=\overset{C}{\underset{j\;=\;1}{\sum }}M_{j}\left(\mu_{j}-\mu \right){\left(\mu_{j}-\mu \right)}^{T},\mu =\frac{1}{C}\overset{C}{\underset{j\;=\;1}{\sum }}\mu_{j} \end{array}$$

(3) $$\begin{array}{r} E=S_{w}^{-1}S_{b} \end{array}$$

The *C-1* eigenvectors of matrix *E* corresponding to the *C* - 1 largest eigenvalues build up the new projection space, denoted by *W*_*opt*_. The original data are projected onto *W*_*opt*_ by the linear transformation:

(4) $$\begin{array}{l} Z=W_{\text{opt}}^{T}X \end{array}$$

*Z*_*ji*_ *(i* = *1, …, M*_*j*_*; j* = *1, …, C)* denotes the projected data. Suppose a test sample with projected value *z*_*test*_, the distance between the test sample and each class is defined as the average Euclidean distance from the test sample to each training sample in the class:

(5) $$D_{\text{j}}=\frac{1}{M_{j}}\sum_{i\;=\;1}^{M_{\text{j}}}\sqrt{(z_{test}-z_{ji}){\left(z_{test}-z_{ji}\right)}^{T}},\;j=1,...,C$$

The test sample is then assigned to the class with the shortest distance (min(*D*_*j*_)).

**Figure A1:**
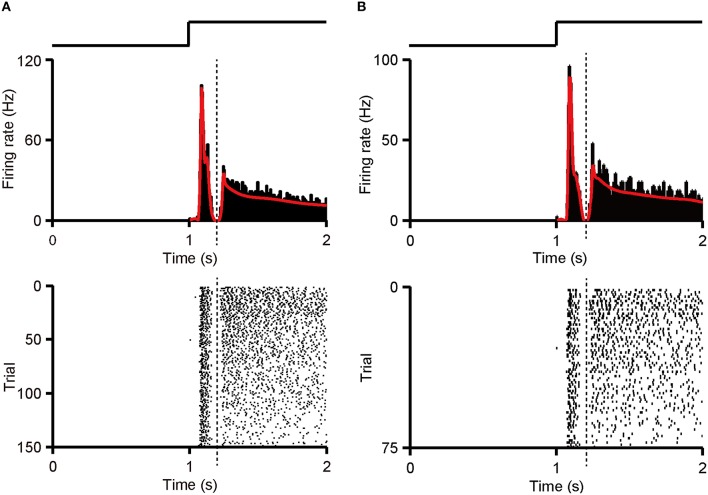
**Separation of first and second response component**. **(A)** PSTH (top panel, bin size = 5 ms) and raster (bottom panel) of an example RGC with dual-peak pattern are shown. PSTH was calculated from whole 150 (50 trials * 3 patterns) response sequences. Dotted line marks the trough in the PSTH. **(B)** PSTH (top panel, bin size = 5 ms) and raster (bottom panel) of the same RGC as in **(A)**. PSTH was calculated from 75 (25 trials * 3 patterns) randomly selected training response sequences. The trough appeared in the PSTH was regarded as the boundary (dotted line). The traces above the PSTH plots in **(A,B)** illustrate the time course of the light stimulation.

In our present study, there were three stimulus-interval patterns to be identified. The procedure of classifying stimulus-pattern-dependent neuronal responses consisted of the following steps: (1) Determining the response features: For each stimulus pattern, the responses from half of the 50 trials were randomly selected as training data, and the remaining 25 responses were used as test data. The PSTH was then calculated based on the 75 training responses (25 repeats * 3 patterns) (Figure A1 in Appendix). The time when the trough appeared in the PSTH was regarded as the boundary of the two response components. First and second component in each training and test response were separated by the identified boundary. From each individual presentation of the light stimuli, the number of spikes and the timing of the first spike relative to the stimulus onset from each response peak were extracted for each cell and employed as the discriminant variables. (2) Calculation of correct classification rate. Following Equations (1–5), each test response was assigned to an estimated stimulus pattern. The estimated result was then compared with the actual stimulus, and the ratio of correct classification was calculated. In our analysis, the classification procedures (1) and (2) described above were independently repeated 10 times, and the final correct classification rate was the averaged ratio obtained over 10 times of training.

**Figure 2:**
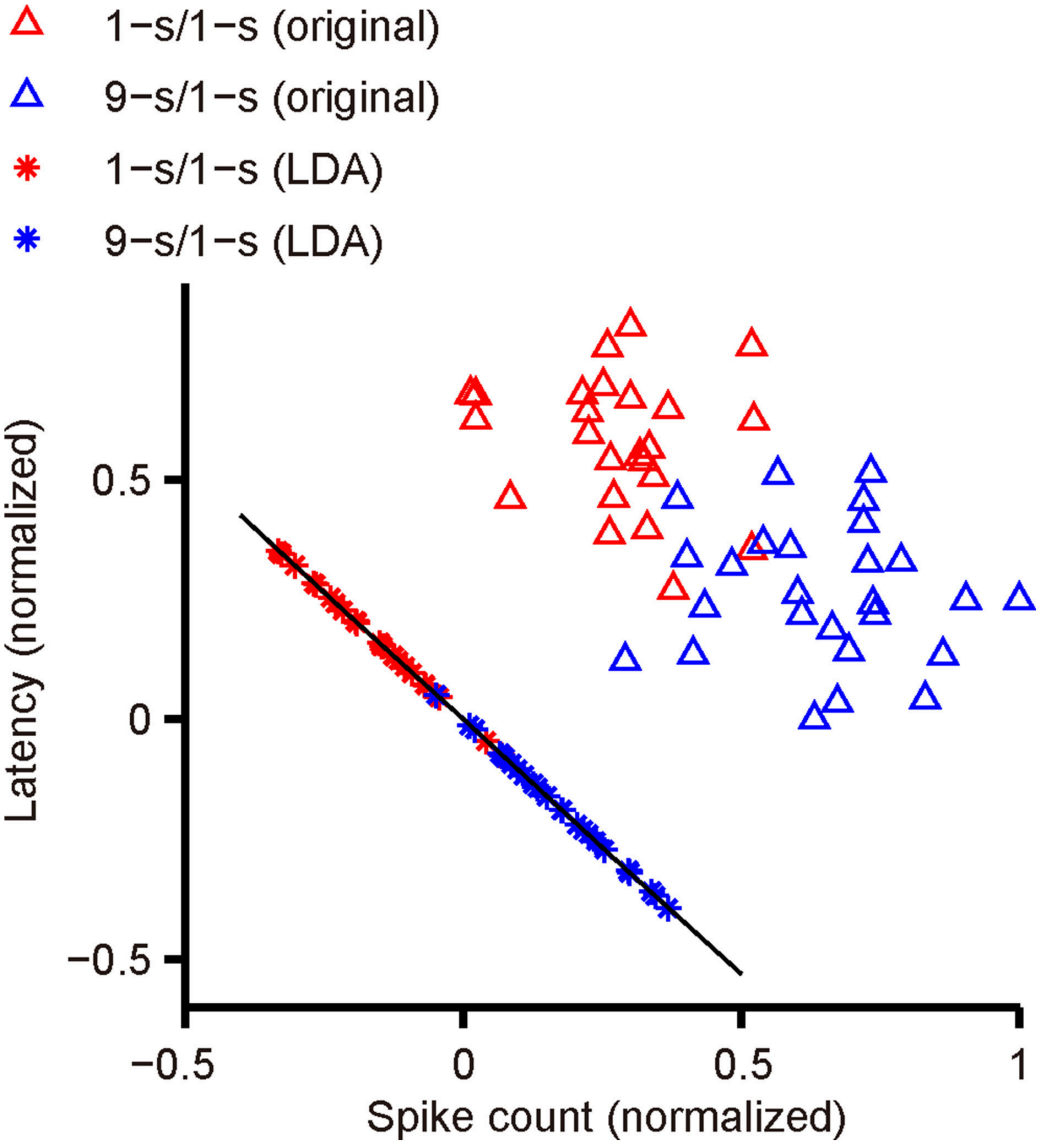
**The separation of two response classes by LDA**. The scatter plot shows the latency and spike count from one response peak of an example RGC with dual-peak pattern. Red triangles indicate latency and spike count during 1-s/1-s (OFF/ON) stimulus pattern (25 trials), and blue triangles indicate latency and spike count during 9-s/1-s (OFF/ON) stimulus pattern (25 trials). The black line indicates the optimal projection direction determined by LDA. The red asterisks are the projections of red triangles to the black line, and the blue asterisks are the projections of blue triangles.

Figure 2 shows an actual example of LDA applied to our data. The scatter plot of latency and spike count from one response peak of an example RGC with dual-peak pattern has been shown. Red triangles indicate latency and spike count during 1-s/1-s (OFF/ON) stimulus pattern (25 trials); blue triangles indicate latency and spike count during 9-s/1-s (OFF/ON) stimulus pattern (25 trials). The optimal projection line (the black line) was (0.696, –0.74). The red and blue asterisks are the projections of red and blue triangles to the black line, respectively. It is shown that most of the training responses in the two classes can be well-separated.

In cases where only one discriminant variable (latency or spike count) was used (**Figure 5**), the eigenvector calculated by the above procedures was 1 (no data projection), then the test response was actually assigned to the class with the shortest distance (min(*D*_*j*_)):

(6) $$D_{\text{j}}=\frac{1}{M_{j}}\sum_{i=1}^{M_{j}}\sqrt{{\left(z_{test}-z_{ji}\right)}^{2}},\text{ j}=1,...C$$

When classification was based on more than one cell (**Figure 7**), linear combinations of the responses features of individual cells were determined. For instance, for the classification based on latency and spike count from both response peaks of the seven-cell group, the optimal linear combinations of 4 × 7 discriminant variables were determined.

LDA was performed using Matlab (version 7.0.0, The MathWorks, Inc., Natick, MA, USA).

## Results

The RGCs studied here responded to light-ON stimuli with two components: one transient component and another following component occurring shortly after the initial one, resulting in two peaks in the PSTH (Figure 1). The second component could be either transient or sustained (Zhou et al., 2007; Yan et al., 2016).

Our experiments were performed on 3 mouse retinas, totally 45 RGCs with ON responses were recorded (including both ON RGCs and ON-OFF RGCs), among which 18 (40%) exhibited dual-peak patterns (5, 6, and 7 cells from retinas #1, #2, and #3, respectively). The receptive fields of these 18 RGCs were also calculated (data not shown). The average major and minor axes of the receptive field center (1 SD of Gaussian) was 121.2 ± 2.9 μm, 100.8 ± 2.2 μm, respectively (Mean ± SEM, *N* = 18).

### Response characteristics of dual-peak RGCs during exposure to light-ON stimuli led by different light-OFF intervals

Response latency and spike count are most common indices to characterize neuronal response (Gollisch and Meister, 2008; Risner et al., 2010; Xiao et al., 2014b). In our present study, response latency was defined as the timing of the first spike relative to the stimulus onset (Gollisch and Meister, 2008). Spike count was defines as the number of spikes in one trial. For dual-peak RGCs, following identification of two response peaks (Materials and Methods), latency and spike count for each peak were measured respectively.

**Figure 3:**
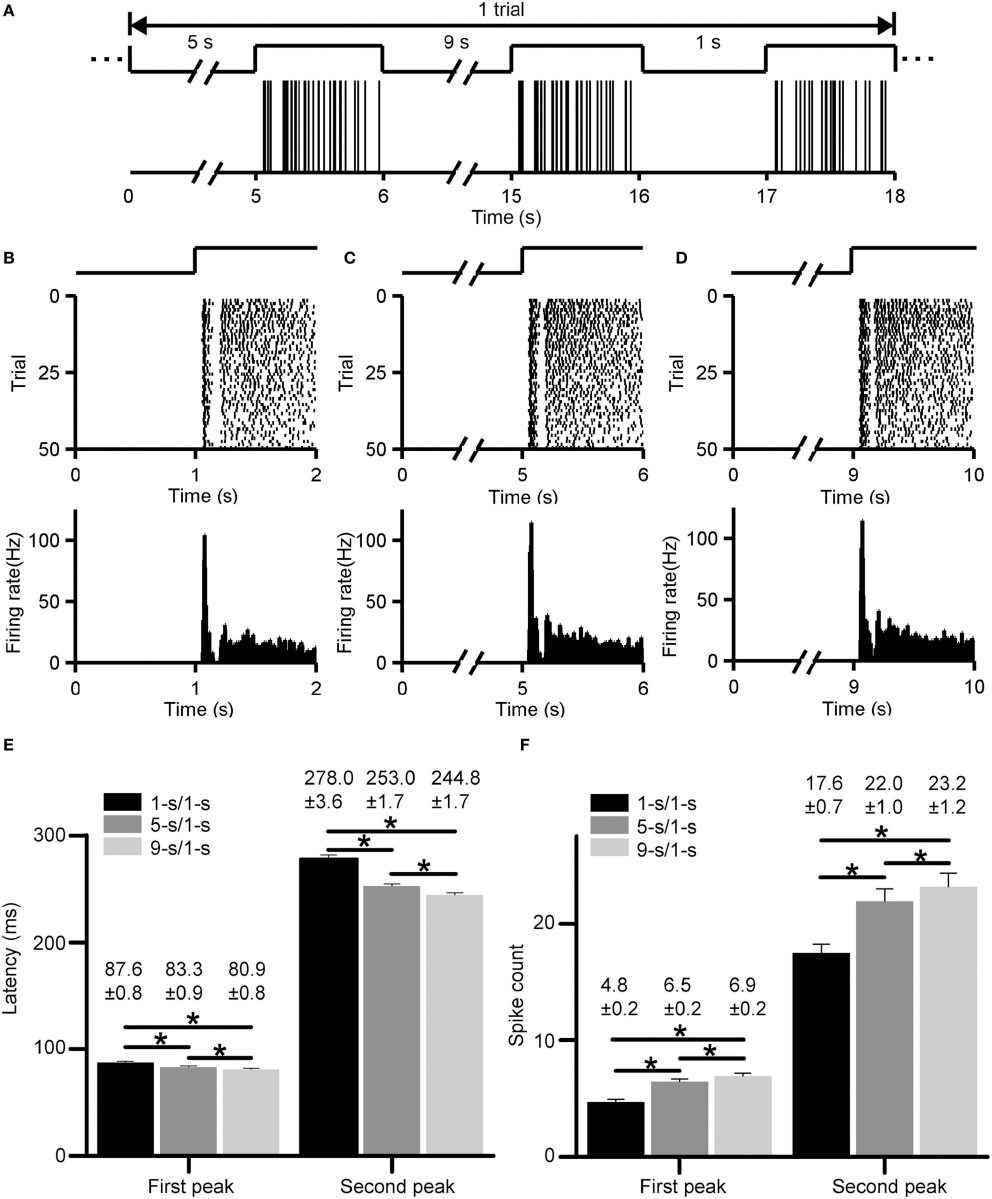
**Responses of an example RGC with dual-peak pattern elicited by light-ON stimuli led by different light-OFF intervals (1, 5, 9 s). (A)** The firing activities of the cell in one trial. The occurrence of each spike is represented by a vertical line. **(B–D)** Raster plot (top panel) and PSTH (bottom panel, bin size = 5 ms) of the cell's responses during 1, 5, and 9-s/1-s (OFF/ON) stimulus patterns, respectively. The traces above the raster plots in **(A–D)** illustrate the time course of the light stimulation. **(E,F)** The average response latencies **(E)** and spike counts **(F)** for the first and second peak of the example RGC, respectively. *N* = 50 trials. Data are presented as mean ± SEM. *p < 0.05, paired *t*-test.

Typical ON responses of an RGC with dual-peak pattern elicited by light-ON stimuli led by various preceding light-OFF intervals (1, 5, 9 s) are plotted in Figure 3. Figure 3A shows the firing activities in one trial, with the occurrence of each spike being represented by a vertical line. Figures 3B–D show the cell's responses during 1, 5, and 9-s/1-s (OFF/ON) stimulus patterns (50 trials), respectively. This example RGC shows dual-peak patterns in exposure to all the three stimulation patterns, suggesting that the emergence of this particular pattern is independent on the stimulus-interval pattern. Average response latencies and spike counts of the two peaks of this example neuron are shown in Figures 3E,F. The response latencies of the first and second peak were shortened significantly when light-OFF interval was increased (Figure 3E, paired *t*-test, *p* < 0.05), while spike counts of both peaks tended to be increased significantly (Figure 3F, paired *t*-test, *p* < 0.05). This was consistent with previous findings in bullfrog retina (Xiao et al., 2014a,b).

**Figure 4:**
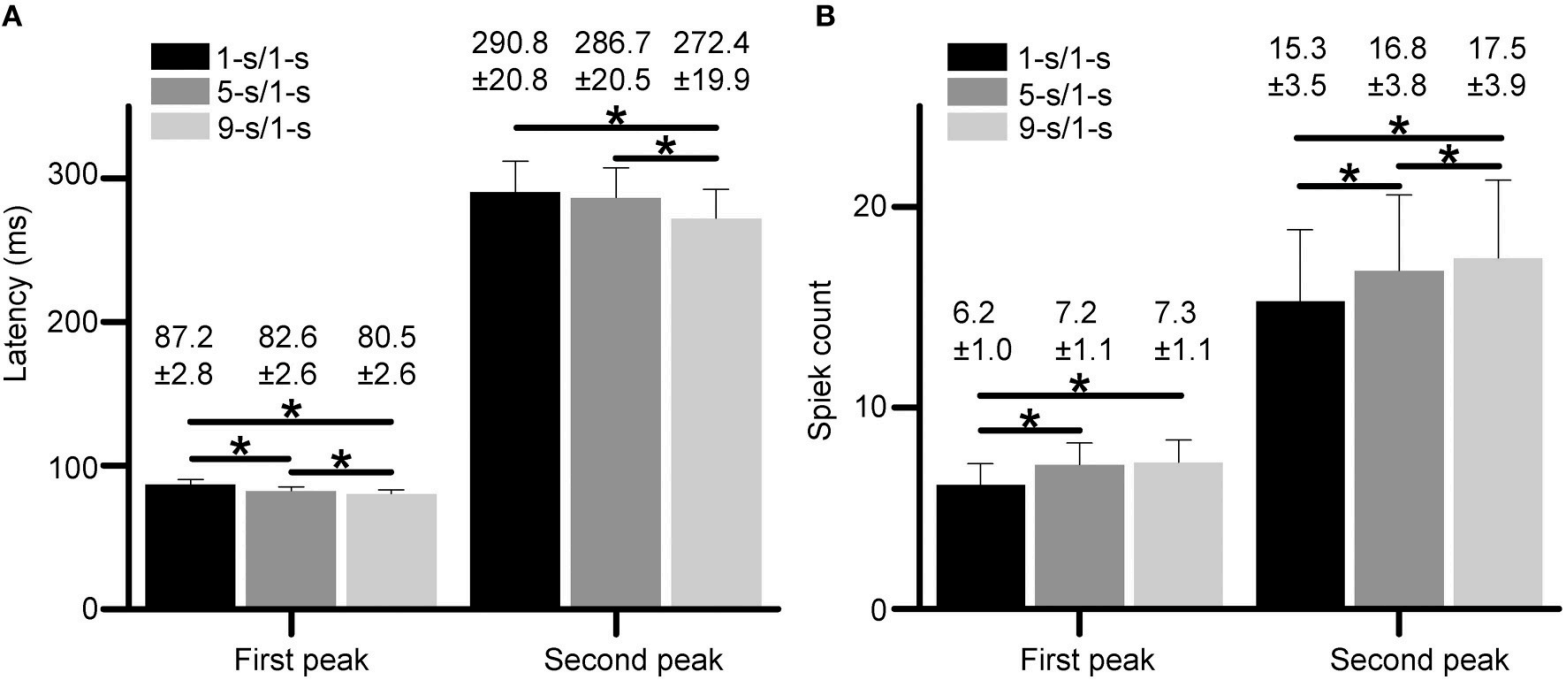
**(A,B)** Statistical results of response latencies **(A)** and spike counts **(B)** for both the first peak and second peak during 1, 5, and 9-s/1-s (OFF/ON) stimulus patterns. *N* = 18 RGCs from three retinas. Data are presented as mean ± SEM. *p < 0.05, paired *t*-test.

Statistical results from 18 RGCs in three retinas show that the latencies for both peaks had small but significant differences among different preceding light-OFF interval groups (Figure 4A, paired *t*-test, *p* < 0.05), with the longest latency for 1-s/1-s (OFF/ON) stimulus pattern, and shortest for 9-s/1-s stimulus pattern. Meanwhile, spike counts for both peaks were also significantly increased with longer preceding light-OFF intervals (Figure 4B, paired *t*-test, *p* < 0.05). The response variabilities of the first and second peak were correlated with the changes of stimuli, suggesting that both peaks might carry information about the stimuli.

### Stimulus identification

To test whether the two peaks in the dual-peak response pattern cooperate to carry stimulus information, we applied LDA method to compare the light-OFF-interval discrimination results based on different features of the RGC responses.

For the pattern discrimination, the following response features from each presentation of the light stimuli were extracted for each cell as discriminant variables: (1) the response latency of the first peak (T1); (2) the number of spikes in the first peak (R1); (3) the response latency of the second peak (T2); (4) the number of spikes in the second peak (R2); (5) the total number of spikes in the response (R_total_). Then these response features could be used by LDA method individually or in linear combinations to discriminate different stimulus-interval patterns that elicited the responses.

**Figure 5:**
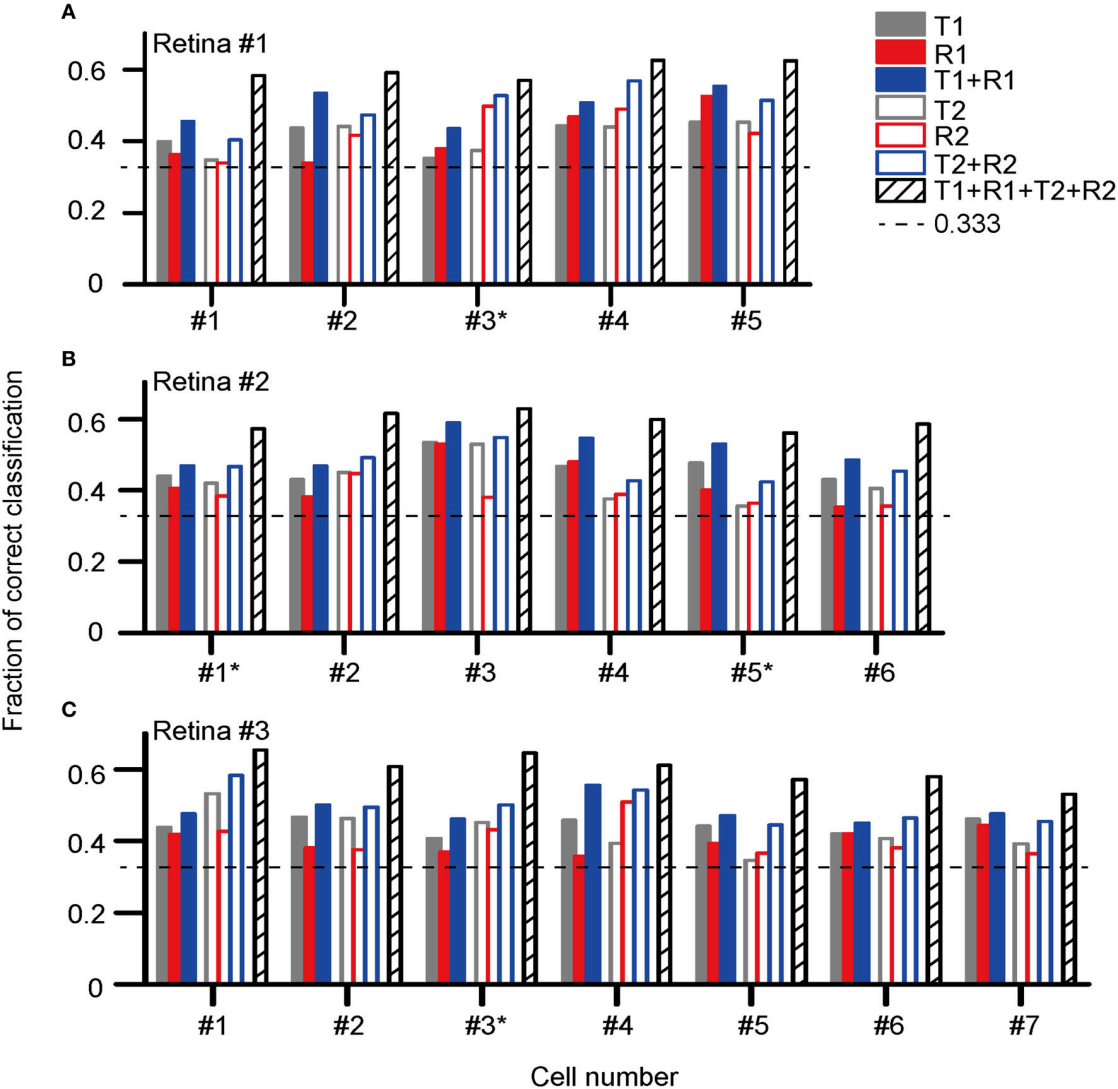
**Correct rates for stimulus pattern discrimination based on single neuron's activity features in (A) retina #1 (five cells, #1–5); (B) retina #2 (six cells, #1–6); (C) retina #3 (seven cells, #1–7)**. The cell label with an asterisk aside indicates this is an ON-OFF RGC. The encoding of dual-peak patterns in ON and ON-OFF RGCs exhibited consistent properties.

Figure 5 presents the classification results for the three retinas recorded (retina #1, #2, and #3), in which the fractions of correctly identified stimuli for each cell based on its response features are plotted. The cell label with an asterisk aside indicates this is an ON-OFF RGC.

The results show that for each cell, the stimulus discrimination based on either latency (T1) or spike count (R1) of the first peak was effective, which allowed stimulus discrimination above the chance level (0.33). By combining T1 and R1 (T1 + R1), the stimulus classification performance was improved, suggesting that stimulus information was carried by both response latency and spike count. Similar to that of the first peak, both the response latency and spike count of the second peak contributed to the information coding. The correct rate of stimulus discrimination was above the chance level while either latency (T2) or spike count (R2) of the second peak was used, and the performance was further improved by using the combination of T2 and R2 (T2 + R2). The best prediction result was obtained for each cell when the response parameters specified for both peaks were taken into account (T1 + R1 + T2 + R2).

**Figure 6:**
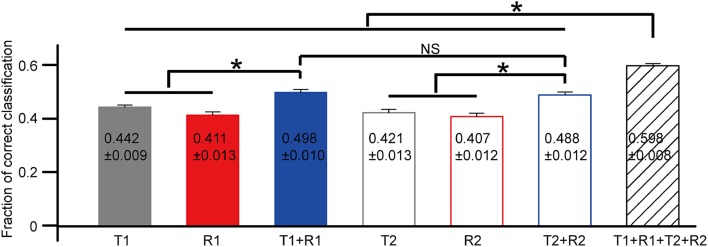
**The comparison of correct rates based on single neuron's activity features**. *N* = 18 RGCs from three retinas. Data are presented as mean ± SEM, with the values being indicated in the bar. *p < 0.05, one-way ANOVA followed by *post hoc* SNK test. NS: not significant.

The statistical results of correct rates based on each selected feature are exhibited in Figure 6 (*N* = 18 cells from three retinas). Data were presented as mean ± SEM. The comparison was performed using one-way ANOVA with *post hoc* Student-Newman-Keuls (SNK) test. For the first peak, the mean correct rate based on the combination of T1 and R1 [C(T1 + R1)] was significantly larger as compared to that of T1 [C(T1)] and R1 [C(R1)] (Figure 6, ANOVA, *p* < 0.05). Similar results were observed for the second peak that C(T2 + R2) was significantly larger than C(T2) and C(R2) (Figure 6, ANOVA, *p* < 0.05). The mean value for C(T1 + R1 + T2 + R2) was the largest, exhibiting significant differences with all the other parameters (Figure 6, ANOVA, *p* < 0.05).

**Figure 7:**
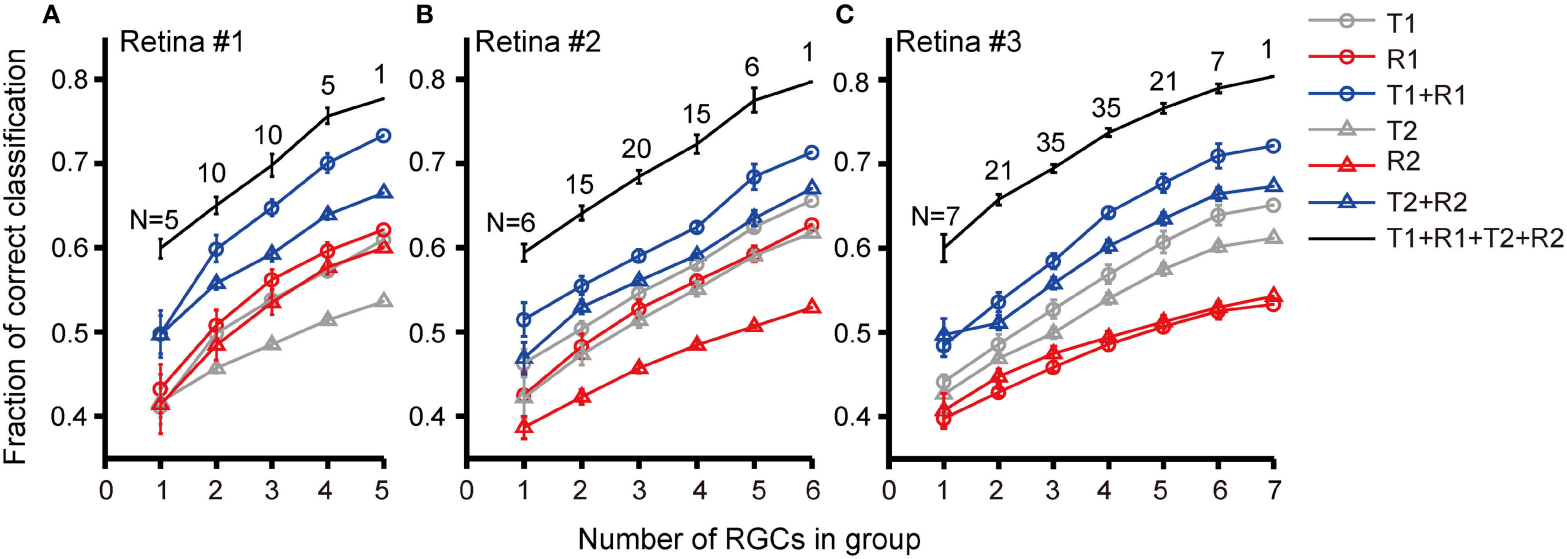
**Correct rates for stimulus pattern discrimination based on population neuron's activity features**. Correct rate was plotted against RGC group size recorded in **(A)** retina #1; **(B)** retina #2; **(C)** retina #3. Data are presented as mean ± SEM. The numbers above the lines indicate the number of groups in each group size.

We further investigated discrimination performance for group of RGCs. The relationship between discrimination performance and RGC group size was exhibited in Figure 7. For retina #1 in which 5 RGCs were recorded, five 1-cell groups, ten 2-cell groups, ten 3-cell groups, five 4-cell groups, and one 5-cell group could be obtained. The average correct rates based on different response features for each group size were compared as shown in Figure 7A. The results exhibited that when the group size was fixed, the correct rate for stimulation identification using combined parameters was higher than that using single parameter [(T1 + R1) vs. T1 or R1; (T2 + R2) vs. T2 or R2], and the best prediction result was obtained using (T1 + R1 + T2 + R2). This was similar to that observed from single cell. Meanwhile, for any selected response features, either being used individually (T1, R1, T2, R2) or in combinations (T1 + R1, T2 + R2, T1 + R1 + T2 + R2), RGC groups exhibited a higher correct rate of pattern discrimination than single RGCs. In addition, the correct rate of RGC group was positively correlated with group size. Though not many RGCs were included in a neuronal group which was due to the limited number of dual-peak cells recorded in one retina, the increase in correct rate of pattern discrimination was obvious. Thus the population activity of RGC group improved neurons' capacity for specific pattern identification. This is consistent with the notion that RGC population activity improves coding efficiency (Fernandez et al., 2000; Schwartz et al., 2012).

Similar results were obtained from the other two retinas (retinas #2 and #3), in which 6 and 7 RGCs with dual-peak patterns were record respectively, as shown in Figures 7B,C.

## Discussion

In the present study, the response features and coding properties of RGCs with dual-peak patterns during exposure to different stimulus-interval patterns were investigated. Our results suggest that the properties of both peaks in the RGCs' light-ON responses can be modulated by different preceding light-OFF intervals (Figure 4). Stimulus discrimination results derived by LDA method demonstrated that both response peaks contributed to stimulus-interval coding, and better discrimination performance was obtained when the response parameters of both peaks were taken into account (Figure 5). These results suggest that both peaks were involved in retinal information coding, and they might carry information complementary to each other.

In a previous study, it was reported that the two peaks in the dual-peak pattern were involved in light intensity discrimination, and the generation of dual-peak pattern was independent on flash intensity and contrast (Thiel et al., 2006). The present study further observed that RGCs showed dual-peak responses during exposure to light-ON stimuli led by different light-OFF intervals, suggesting that the emergence of this particular pattern was also independent on the preceding light-OFF interval. Thus the occurrence of dual-peak response might be stimulus independent, with its properties modulated by different stimulus parameters. According to one of our previous works, the dual-peak response might originate from the convergence of two pathways related to the short-latency response and long-latency response respectively (Yan et al., 2016).

### The reliable encoding of stimulus-interval pattern by changes in the RGC response

Visual stimulation contains many important features, such as stimulus intensity, contrast, and duration, etc. Previous studies showed that both peaks in dual-peak pattern contributed to light intensity coding (Thiel et al., 2006). In the present study, we focused on the stimulus-interval-dependent response changes and information coding for dual-peak cells. Here only the encoding of different preceding light-OFF intervals was investigated, because the dual-peak responses recorded were mostly ON responses, observed in ON RGCs and light-ON part of ON-OFF RGCs (Soucy et al., 1998; Yan et al., 2016). In previous study, it has been reported that the latency and firing rate of ON responses were modulated by preceding light-OFF intervals, but independent on the following light-ON durations (Xiao et al., 2014b). The interval was set within the range of seconds. In natural environments and our daily life, many stimuli last for the order of second, for example, the visual distress signal (SOS) consists of three short, three long, and three short flashes of light, with the long flashes lasting for seconds. The red beacons at the rear of a train also flash in seconds to signal an oncoming train.

Meanwhile, RGCs' responses also depend on many other parameters of stimulus, such as intensity, contrast and spatial structure and so on. In the present study, all stimulus parameters, except the preceding interval, were kept constant during experiment. In this way, the changes of RGC responses were only stimulus-interval related, and the reliable encoding of the preceding interval can be guaranteed. However whether the changes of other stimulus parameters would influence the interval-related response changes remains an interesting topic and needs further investigation.

### Rate coding, temporal coding, and population coding

Whether neurons use a rate code or a temporal code has been a classic concern in visual neuroscience. It has been a long time that the importance of rate coding prevails. However, increasing evidence has shown that the temporal properties (such as response latency, special temporal pattern, and inter-spike interval) of the neuronal firing activity also contain information (Berry et al., 1997; Lesica and Stanley, 2004; Greschner et al., 2006; Gollisch and Meister, 2008; Kretschmer et al., 2012; Xiao et al., 2014b), demonstrating the importance of temporal coding for neural information. Besides, RGCs do not fire independently, an ensemble of RGCs can work in concerted ways to encode information more reliably and efficiently (Pillow et al., 2008; Jing et al., 2010; Li and Liang, 2013).

In our present study, temporal coding and rate coding were partly (but not fully) redundant to each other, given that the correct rate of stimulation pattern discrimination when response features (first spike timing and spike count) were considered in combination C(T + R) was smaller than the sum of correct rate C(T) and C(R). This might be due to that the changes of latency and firing rate were correlated, i.e., an increased latency was normally accompanied by a decreased firing rate, and vice versa. Negative correlation between latency and firing rate was also a common observation throughout the visual system, which was reported to be related to the strength of stimuli (Maunsell et al., 1999; Reich et al., 2001; Risner et al., 2010; Xiao et al., 2014a,b). Thus it is presumable that there might be some common mechanism contributing to the changes of latency and firing rate (Cleland and Enroth-Cugell, 1970; Lennie, 1981). In the present study, the changes of latency and firing rate might due to the changes of sensitivity of RGCs which were modulated by different preceding light-OFF intervals.

However, latency and spike count were not fully dependent on each other, therefore, the combination of these two aspects brought a small (5~10% percent of increase) but significant enhancement in stimulus identification performance (Figure 6). And, the increase was explainable: reliable discrimination of different stimulus patterns depends on the reliable stimulus-response relationship. Due to the noise in the RGC's response, the stimulus-related changes of single parameter might sometimes be contaminated, leading to inaccurate stimulus identification. Since latency and firing rate both exhibited stimulus-related changes, the combination of the two parameters might provide a more reliable stimulus-response relationship, thus performing better in stimulus identification. These results are well consistent with previous findings (Fernandez et al., 2000).

Besides, it was also observed that the coding performance of a neuron group was related to the number of neurons in the group. The classification performance was improved while the group size was increased, which means that more reliable information about the stimulus features could be deduced from spatio-temporal response patterns of retinal ganglion cell population (Schwartz et al., 2012). These results demonstrate that the three suggested types of neural information coding (rate coding, temporal coding, and population coding) are all involved in encoding the different stimulus-duration patterns.

### The firing activities in the two response peaks cooperate in information coding

Discrimination results show that estimation performance was improved when response features of two response peaks were considered in combination (T1 + R1 + T2 + R2) as compared to that when response features of a single peak (T1 + R1 or T2 + R2) were considered. Discrimination of stimulus patterns depends on the reliable differences in the responses elicited by different stimulations. Since response features of both peaks exhibited stimulus-related changes, thus taking both peaks into consideration would better reveal the differences in the neural responses, which then lead to better performance in discrimination.

On the other hand, although the two response peaks cooperate to improved stimulus identification, the information carried by the two peaks are redundant, which could be simply obtained from the result that C(T1 + R1 + T2 + R2) was smaller than the sum of correct rate C(T1 + R1) and C(T2 + R2). Redundancy introduced by population code has been widely investigated, and has been thought of as a way to improve the reliability in information transmission (Puchalla et al., 2005). Here we report the improvement in the stimulus discrimination brought by the temporal pattern of RGC responses, in the presence of redundancy. Redundancy compromises efficiency, with more spikes being used to represent the same information (Puchalla et al., 2005), therefore, how retina trades off redundancy and efficiency still remains an interesting topic to explore (Tkačik et al., 2010; Doi et al., 2012; Palmer et al., 2015).

From a recent work in our lab, it was found that the two response peaks in the dual-peak pattern may originate from two different pathways related to brisk response and sluggish response respectively (Yan et al., 2016). Our present study added to the point by showing that taking dual-peak pattern as two discrete spike events instead of as a single event led to better discrimination performance: C(T1 + R1 + T2 + R2) > C(T1 + R_total_) (data not shown). It has been identified that, brisk cells and sluggish cells serve different functions in perception. Brisk cells are highly sensitive to stimulus contrast, while sluggish cells are quite selective for particular features, including local edge, motion direction (Troy and Shou, 2002; Dhingra et al., 2003). So could it possible that the two peaks in dual-peak response may also encode different information about the stimulus, and work in different stimulus conditions, this needs further investigation.

## Author contributions

RY and PL designed experiments. RY and HG performed experiments. RY and PZ performed data analysis. RY, PZ, and PL wrote the paper.

## Funding

This work was supported by grants from National Natural Science Foundation of China (No. 31471054, PL; No. 61375114, PZ).

### Conflict of interest statement

The authors declare that the research was conducted in the absence of any commercial or financial relationships that could be construed as a potential conflict of interest.
